# CXCL12/CXCR4-Mediated Procollagen Secretion Is Coupled To Cullin-RING Ubiquitin Ligase Activation

**DOI:** 10.1038/s41598-018-21506-7

**Published:** 2018-02-22

**Authors:** Susan Patalano, José Rodríguez-Nieves, Cory Colaneri, Justin Cotellessa, Diego Almanza, Alisa Zhilin-Roth, Todd Riley, Jill Macoska

**Affiliations:** 1 Department of Biology, University of Massachusetts Boston, Boston, United States; 2 Center for Personalized Cancer Therapy, University of Massachusetts Boston, Boston, United States

## Abstract

Tissue fibrosis is mediated by the actions of multiple pro-fibrotic proteins that can induce myofibroblast phenoconversion through diverse signaling pathways coupled predominantly to Smads or MEK/Erk proteins. The TGF**β**/TGF**β**R and CXCL12/CXCR4 axes induce myofibroblast phenoconversion independently through Smads and MEK/Erk proteins, respectively. To investigate these mechanisms at the genetic level, we have now elucidated the TGF**β**/TGF**β**R and CXCL12/CXCR4 transcriptomes in human fibroblasts. These transcriptomes are largely convergent, and up-regulate transcripts encoding proteins known to promote myofibroblast phenoconversion. These studies also revealed a molecular signature unique to CXCL12/CXCR4 axis activation for COPII vesicle formation, ubiquitination, and Golgi/ER localization/targeting. In particular, both CUL3 and KLHL12, key members of the Cullin-RING (CRL) ubiquitin ligase family of proteins involved in procollagen transport from the ER to the Golgi, were highly up-regulated in CXCL12-, but repressed in TGF**β**-, treated cells. Up-regulation of CUL3 and KLHL12 was correlated with higher procollagen secretion by CXCL12-treated cells, and this affect was ablated upon treatment with inhibitors specific for CXCR4 or CUL3 and repressed by TGF**β**/TGF**β**R axis activation. The results of these studies show that activation of the CXCL12/CXCR4 axis uniquely facilitates procollagen I secretion through a COPII-vesicle mediated mechanism to promote production of the ECM characteristic of fibrosis.

## Introduction

Myofibroblast phenoconversion occurs in response to a pro-fibrotic stimulus, canonically TGF**β** signaling through TGF**β**RI (ALK5) and TGF**β**RII^1,2^. Recent efforts by our group to elucidate the mechanisms through which CXCL12 contributes to fibrosis reveal that CXCL12 signals through the CXCR4 G-protein coupled receptor to initiate myofibroblast phenoconversion, independently of TGFβ^3^. Although both the CXCL12/CXCR4 and TGF**β**/TGF**β**R axes can activate MAP Kinase signaling, TGF**β** promotes myofibroblast phenoconversion in a Smad-dependent manner, whereas CXCL12/CXCR4 accomplishes this by transactivating EGFR and promoting downstream MAPK signaling. Both CXCL12- and TGF**β** - mediated signaling results in transcriptional activation of the COL1A1 and COL1A2 gene promoters, COL1A1 and COL1A2 gene transcription, and collagen 1 protein production, which is a hallmark of myofibroblast phenoconversion and ECM deposition characteristic of tissue fibrosis^2,4,5^.

In an effort to understand how both the TGF**β**/TGF**β**R and CXCL12/CXCR4 signaling axes promote the transcription of genes encoding ECM proteins, we have now elucidated and compared the transcriptomes induced by these two pathways in human prostate fibroblasts. These studies show that the TGF**β/**TGF**β**R - and CXCL12/CXCR4-mediated transcriptomes are largely convergent, and include transcripts encoding proteins known to promote myofibroblast phenoconversion and extracellular matrix (ECM) accumulation, as well those encoding proteins coupled to DNA/RNA synthesis and regulation; cell division; respiration and energy metabolism; and protein synthesis and degradation. These studies also revealed a molecular signature unique to CXCL12/CXCR4 axis activation tied to COPII vesicle formation, ubiquitination, and Golgi/ER localization/targeting. COPII vesicles are required for the transport of large cargo, including procollagen proteins, from the endoplasmic reticulum (ER) to the Golgi apparatus. Therefore, activation of the CXCL12/CXCR4 axis promotes collagen gene transcription and uniquely facilitates procollagen I secretion, thereby promoting production of the ECM characteristic of tissue fibrosis.

## Results

### The CXCL12/CXCR4- and TGFβ/TGFβR-Signaling Axes Induce Similar Transcriptomes

**Figure 1:**
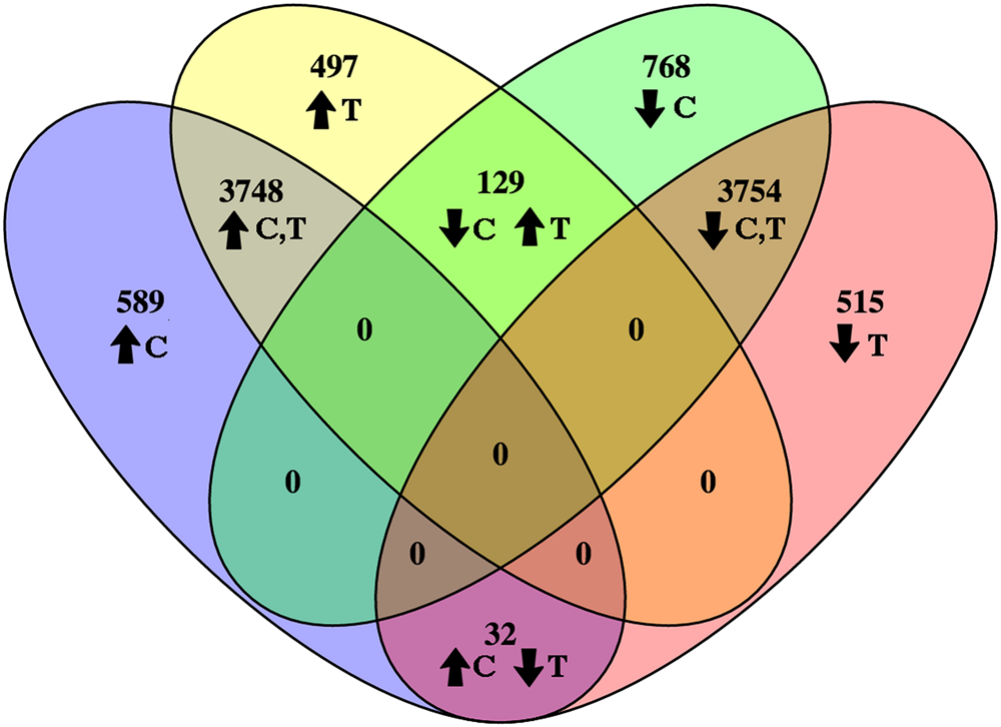
Venn Diagram of CXCL12- and TGFβ-Induced Transcriptomes. EdgeR analysis of gene transcripts differentially expressed consequent to CXCL12- or TGFβ –treatment is expressed as a Venn diagram. Of the total 10,032 transcripts induced by CXCL12 or TGFβ, 7502 (75%) transcripts were similarly up (**↑**C, T) or down (**↓**C, T) - regulated by CXCL12 or TGFβ; 1012 (10%) were differently expressed consequent to TGFβ treatment only (indicated as **↑**T or **↓**T), 1357 (13%) by CXCL12 treatment only (indicated as **↑**C or **↓**C), and 161 (2%) were differentially regulated by CXCL12 and TGFβ (indicated as **↓**C **↑**T or **↑**C **↓**T). Thus, the transcriptional molecular signature significantly induced or repressed by CXCL12 and TGFβ were 75% similar and 25% dissimilar.

These studies utilized human N1 cells which were derived from a stromal nodule of benign prostatic hyperplasia, exhibit a fibroblastic morphology, express fibroblastic markers vimentin and calponin, and demonstrate secretion and proliferation profiles consistent with aging primary prostate fibroblasts^6^,^7^. Analysis of RNASeq data revealed that a total of 10,032 transcripts were induced by CXCL12 or TGF**β** compared to vehicle controls (Supplementary Dataset). Of these, 7502 (75%) transcripts were similarly up- or down-regulated by CXCL12 or TGF**β**, 1012 (10%) were differently expressed consequent to TGF**β** treatment only, 1357 (13%) by CXCL12 treatment only, and 161 (2%) were differentially regulated by CXCL12 and TGF**β** (Fig. 1). Thus, the transcriptional molecular signature significantly induced or repressed by CXCL12 and TGF**β** were 75% similar and 25% dissimilar.

### Gene Ontology Reveals a CXCL12-mediated ECM, ER to Golgi, and Ubiquitination Signatures

**Figure 2:**
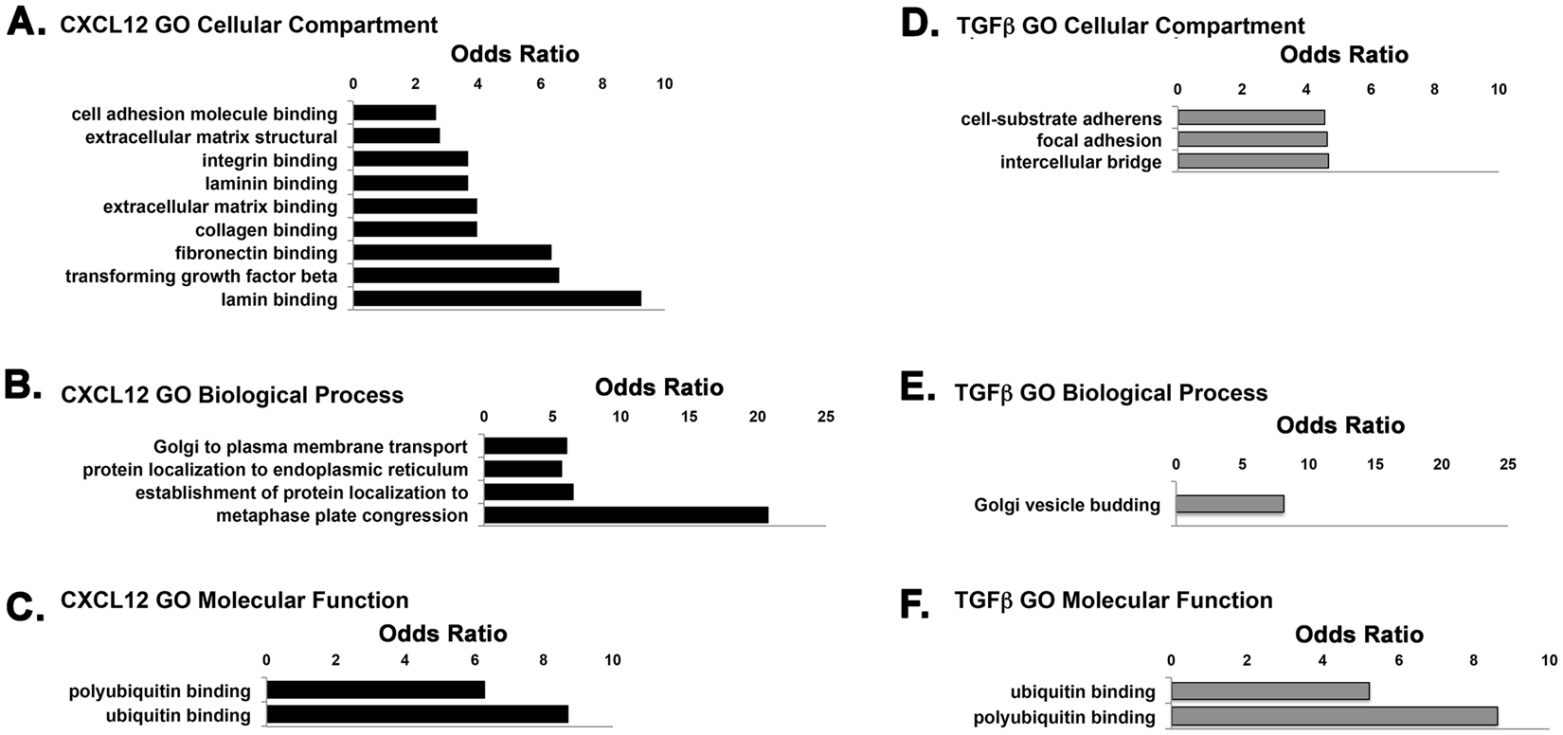
Gene Ontology Analysis of CXCL12- and TGFβ-Induced Transcriptomes. Log odds ratios indicate the relative abundance of Gene Ontology (GO) terms compared to random distribution as categorized by cellular compartment (**A**,**D**), biological process (**B**,**E**) or molecular function (**C**,**F**). Terms related to extracellular matrix and matrix components in the GO cellular compartment category were significantly higher (a significantly higher number of genes related to these terms in this category were up-regulated) for CXCL12-treated (**A**) than TGFβ-treated (**D**) cells, as were biological process terms for Golgi and ER localization and transport (**B**,**E**) and molecular function terms for ubiquitin and polyubiquitin binding (**C**,**F**). The complete log odds ratio analysis of Gene Ontology terms is provided in Supplementary File.

Calculation of the Log odds ratio (LR) revealed the relative abundance of particular Gene Ontology terms compared to random distribution. LR calculation for GO cellular component demonstrated several differences between CXCL12- and TGF**β**-treated cells. Up-regulation of respiratory chain complexes, protein synthesis and degradation, and cell division were predominant in the TGF**β**-mediated signature, whereas cellular signaling and ECM component binding were prevalent in the CXCL12-mediated signature (Supplementary File and Fig. 2A). GO biological process analysis was heavily weighted towards DNA synthesis and mitosis in the TGF**β**-mediated signature. This was less evident in the CXCL12-mediated signature, which was more diverse and included protein synthesis, protein targeting, and localization to the ER and Golgi (Supplementary File and Fig. 2B). GO molecular function analysis showed that proteins encoded by genes regulated by CXCL12 and TGF**β** were coupled to DNA/RNA synthesis and regulation; respiration and energy metabolism; protein synthesis and degradation; and ubiquitination (Supplementary File and Fig. 2C). These categories were predominant in both CXCL12- and TGF**β**-treated cells, though particular categories were not equivalent in each signature. For example, the TGF**β** signature comprised a greater number of genes encoding proteins involved in NADH dehydrogenase metabolism, hence, respiration and energy metabolism, than the CXCL12 signature, whereas the CXCL12 signature demonstrated a greater number of genes encoding proteins coupled to ubiquitination /polyubiquitination (Supplementary File and Fig. 2C**)**.

**Figure 3:**
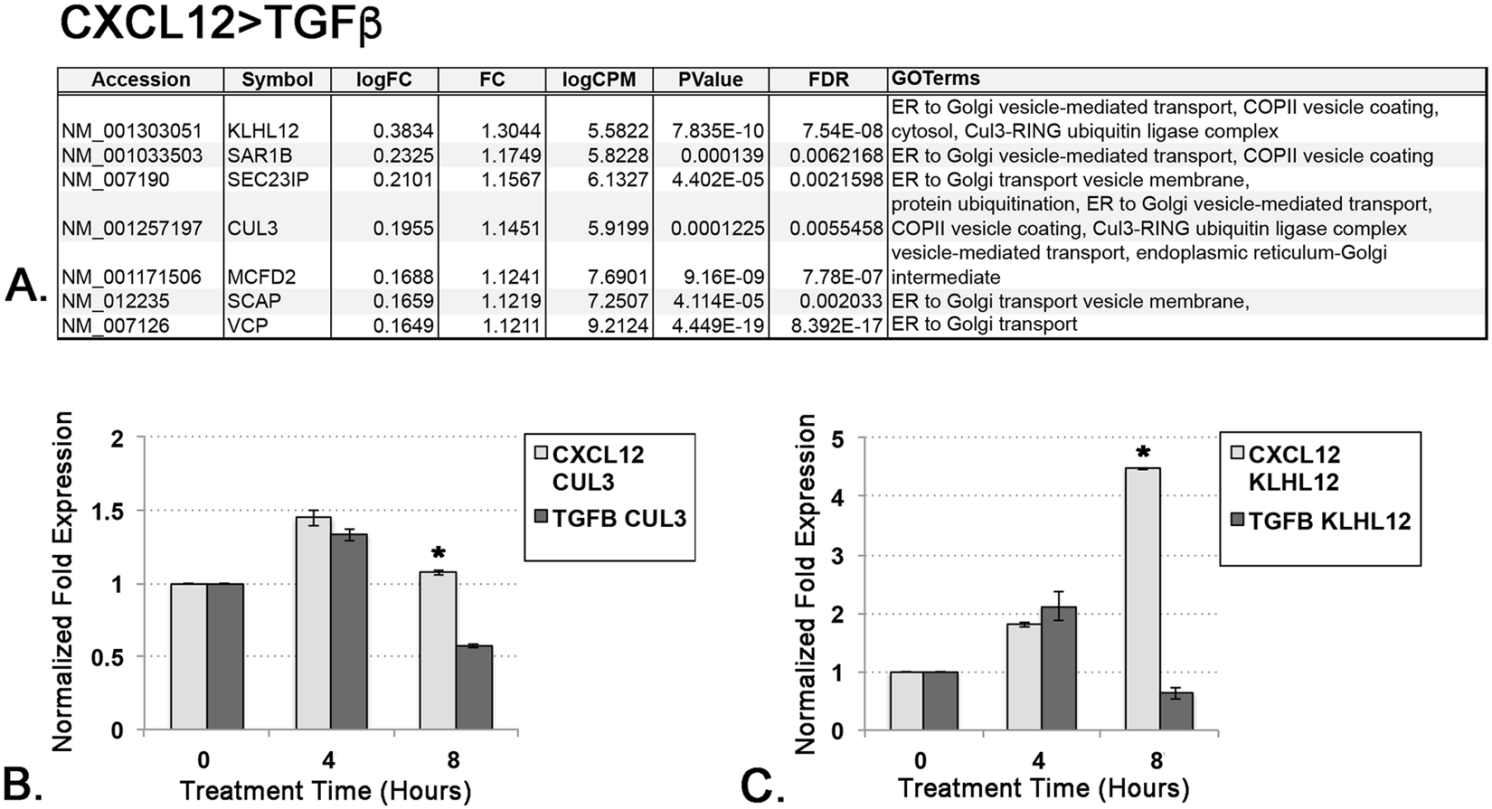
CXCL12-Treated Cells Demonstrate Significant Up-Regulation of Transcripts Encoding COPII Vesicle Proteins. (**A**) Table of GO terms associated with specific genes encoding proteins that function in ER to Golgi transport that were significantly up-regulated by CXCL12, but not TGFβ, treatment. All of these genes encode proteins that comprise multi-subunit RING-finger type 3 Cullin-RBX E3 structures which, upon the addition of KLHL12, forms COPII vesicles essential for procollagen secretion. The gene Accession numbers and Symbols are as indicated. LogFC (log fold change) and FC (fold change) refer to CXCL12 > TGF**β** after normalization to vehicle; logCPM = log counts per million; FDR = false discovery rate. (**B**,**C**) Graphs demonstrating averages of triplicate measures of normalized (to vehicle) fold expression of CUL3 (**B**) and KLHL12 (**C**) in CXCL12- or TGF**β** -treated cells by qRT-PCR analysis confirm that both genes were more robustly transcriptionally induced by CXCL12 than TGF**β**, particularly at 8 hours treatment (* p < 0.05).

**Figure 4:**
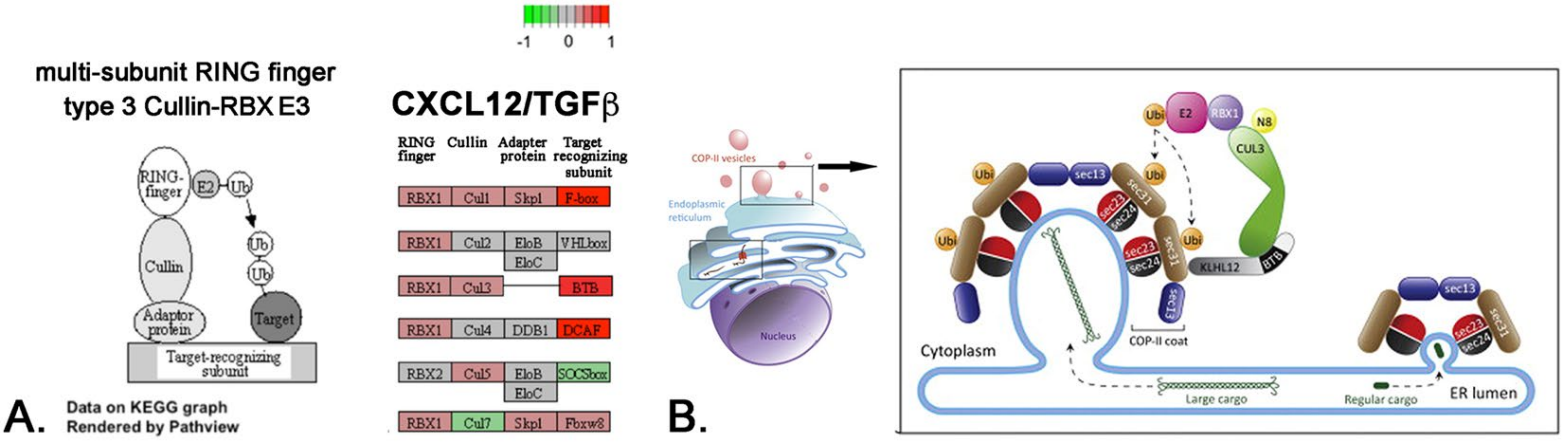
CXCL12 Transcriptionally Up-Regulates Cullin-RING Ubiquitin Ligases. (**A**) KEGG Pathway hsa04120 illustration (left) of multi-subunit RING-finger type 3 Cullin-RBX E3 ubiquitin ligase structure and of differential expression of specific genes in this complex (right). Genes significantly up-regulated consequent to CXCL12 treatment in red, up-regulated consequent to TGF**β** treatment in green, not differentially expressed in gray (arbitrary scale indicates extent of differential expression). (**B**) Illustration of COPII vesicle components which includes proteins encoded by several genes up-regulated consequent to CXCL12 but not TGF**β** treatment. Modified from^13^ and used with permission from Elsevier Limited (VAT number GB 494 6272 12).

The ECM signature evident in the transcriptomes of both CXCL12- and TGF**β**-treated cells was consistent with the known pro-fibrotic functions of these proteins. However, the Golgi transport/endoplasmic reticulum and ubiquitination/polyubiquitination signatures that emerged predominantly in the transcriptome of CXCL12-treated cells were intriguing. A search of GO terms identified 7 genes – *KLHL12*, *SAR1B*, *SEC23IP*, *CUL3*, *MCFD2*, *SCAP*, and *VCP* – encoding proteins that function in ER to Golgi transport that were significantly expressed at higher levels by CXCL12- compared to TGF**β**- treated cells (Fig. 3A). KEGG Pathway analysis revealed that several of these differentially expressed genes encoded proteins that are part of the multi-subunit RING-finger type 3 Cullin-RBX E3 (Fig. 4A). Cullin proteins are molecular scaffolds vital to the assembly of Cullin-RING ubiquitin ligases (CRLs), a class of E3 ubiquitin ligases that facilitate the covalent attachment of ubiquitin moieties to target proteins, leading to ubiquitin-mediated proteolysis and/or regulatory events. The target substrate of the RBX1-CUL3-KLHL12 CRL is SEC31, a protein found on the outside coat of COPII vesicles, which traffic proteins destined for extracellular secretion from the ER to the Golgi. Monoubiquitination of SEC31 by CUL3-KLHL12 is required for the formation of larger COPII vesicles capable of accommodating large cargo such as procollagen, a molecule up to 400 nm in length^8^ (Fig. 4B). As seen in Fig. 3A,transcripts for genes encoding the CUL3, KLHL12, and SEC23 proteins were significantly expressed at higher levels by CXCL12- compared to TGF**β**- treated cells. Other genes preferentially up-regulated by the CXCL12/CXCR4 axis and associated with COPII vesicle-mediated ER-to-Golgi protein secretion include SCAP, which binds to COPII vesicles^9^; SAR1B, a guanine exchange factor that assembles the SEC23-SEC24 COPII vesicle subunits^10^, MCFD2, a calcium-dependent protein that functions to capture COPII vesicle cargo^10^, vasolin-containing protein (VCP), which assists with protein ubiquitination and protein secretion by COPII vesicles^11,12^ (Fig. 3A). qRT-PCR studies focused on two of these genes, KLHL12 and CUL3, validated their differential gene expression in CXCL12- and TGF**β**-treated cells (Fig. 3B). Based on these data, additional experiments designed to investigate the potential role of CXCL12/CXCR4-mediated induction of COPII vesicle components and procollagen secretion were pursued.

### Activation of the TGFβ/TGFβR Axis Represses E3 Ubiquitin Ligase Component Expression

**Figure 5:**
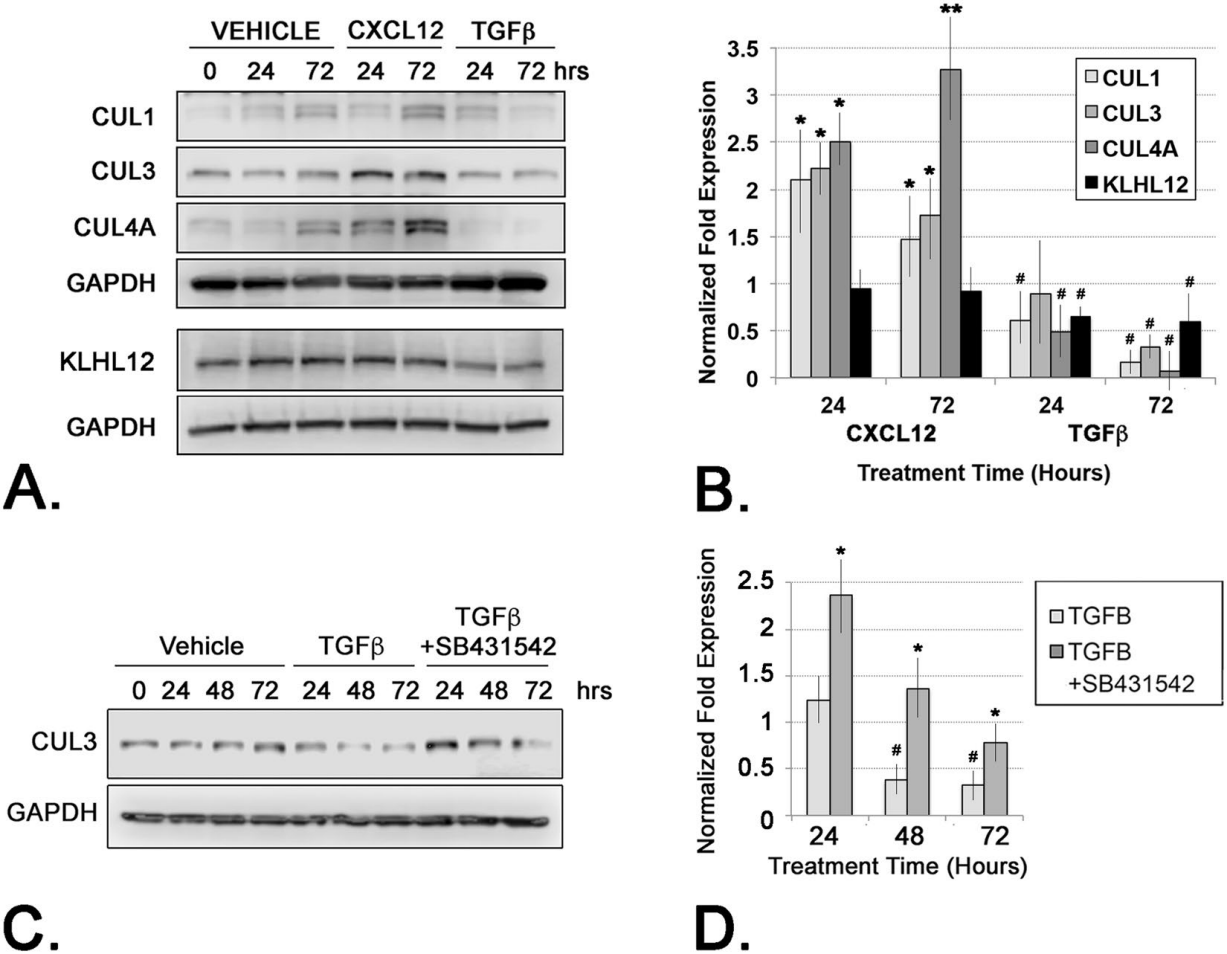
CXCL12 Up-Regulates RING-finger Type 3 Cullin-RBX E3 Ubiquitin Ligase and COPII Vesicle Proteins. (**A**) Immunoblot of protein lysates from cells treated with vehicle, CXCL12 or TGF**β** and probed for CUL1, CUL3, CUL4A, KLHL12 and GAPDH (as loading control) proteins. One filter was stripped and re-probed sequentially for the CUL and GAPDH proteins examined; a separate filter was stripped and re-probed for KLHL12 and GAPDH. (**B**) Graph of densitometric analysis of replicate immunoblots of (**A**) CXCL12 significantly up-regulated CUL1, CUL3 and CUL4A proteins at 24 and 72 hrs treatment compared to vehicle (**p < 0.001; *p < 0.05) whereas these same proteins were down-regulated below basal levels by TGF**β** compared to vehicle (^#^p < 0.05). KLHL12 protein was significantly down-regulated (^#^p < 0.05) in TGF**β**-treated, but not CXCL12-treated, cells. (**C**) Immunoblot of protein lysates from cells treated with vehicle, TGF**β** or the small molecule TGF**β**R1 inhibitor SB431542 + TGF**β**, and probed for CUL3 or GAPDH (as loading control) proteins. (**D**) Graph of densitometric analysis of replicate immunoblots as shown in (**C**) CUL3 protein expression is significantly repressed in TGF**β** -treated cells; this repression is lifted upon co-treatment with SB431542 (^#^ or *p < 0.05 compared to vehicle-treated cells). Graphs represent averaged measures of triplicate experiments.

Immunoblot analyses confirmed that CUL3 protein levels paralleled those of RNA transcript levels and were expressed at significantly higher levels in CXCL12-treated compared to TGF**β** -treated cells (Fig. 5A,B). CUL1 and CUL4A protein levels were also significantly up-regulated by activation of the CXCL12/CXCR4, but remained at basal or below basal levels upon activation of the TGF**β**/TGF**β**R, axis (Fig. 5A,B). RNASeq and qRT-PCR analysis demonstrated significantly higher levels of KLHL12 transcript consequent to CXCL12-, but not TGF**β**-, treatment (Fig. 3A,C). Moreover, TGF**β** treatment significantly repressed KLHL12 protein expression below basal levels, whereas CXCL12 treatment had no effect on KLHL12 protein expression (Fig. 5A,B).

The repression of KLHL12 protein expression consequent to activation of the TGF**β**/TGF**β**R axis suggested that this axis might negatively regulate cellular levels of the cullin-RING ubiquitin ligase (CRL) complex associated with procollagen secretion^13^. To investigate this, cells were treated with TGF**β** in the presence of absence of the TGF**β**R1 -specific small molecular inhibitor, SB431542. As shown in Fig. 5C and quantitated in Fig. 5D,treatment with TGF**β** repressed CUL3 protein expression below those of vehicle-treated cells, consistent with data shown in Fig. 5A,B. The observed TGF**β**-mediated repression of CUL3 protein expression was rescued upon co-treatment with the TGF**β**R1-specific small molecular inhibitor, SB431542 (Fig. 5C,D). This combined gene and protein expression data is consistent with a mechanism whereby, unlike activation of the CXCL12/CXCR4 axis, activation of the TGF**β/**TGF**β**R axis represses E3 ubiquitin ligase component expression.

### Activation of the CXCL12/CXCR4-Axis Promotes Procollagen Secretion

**Figure 6:**
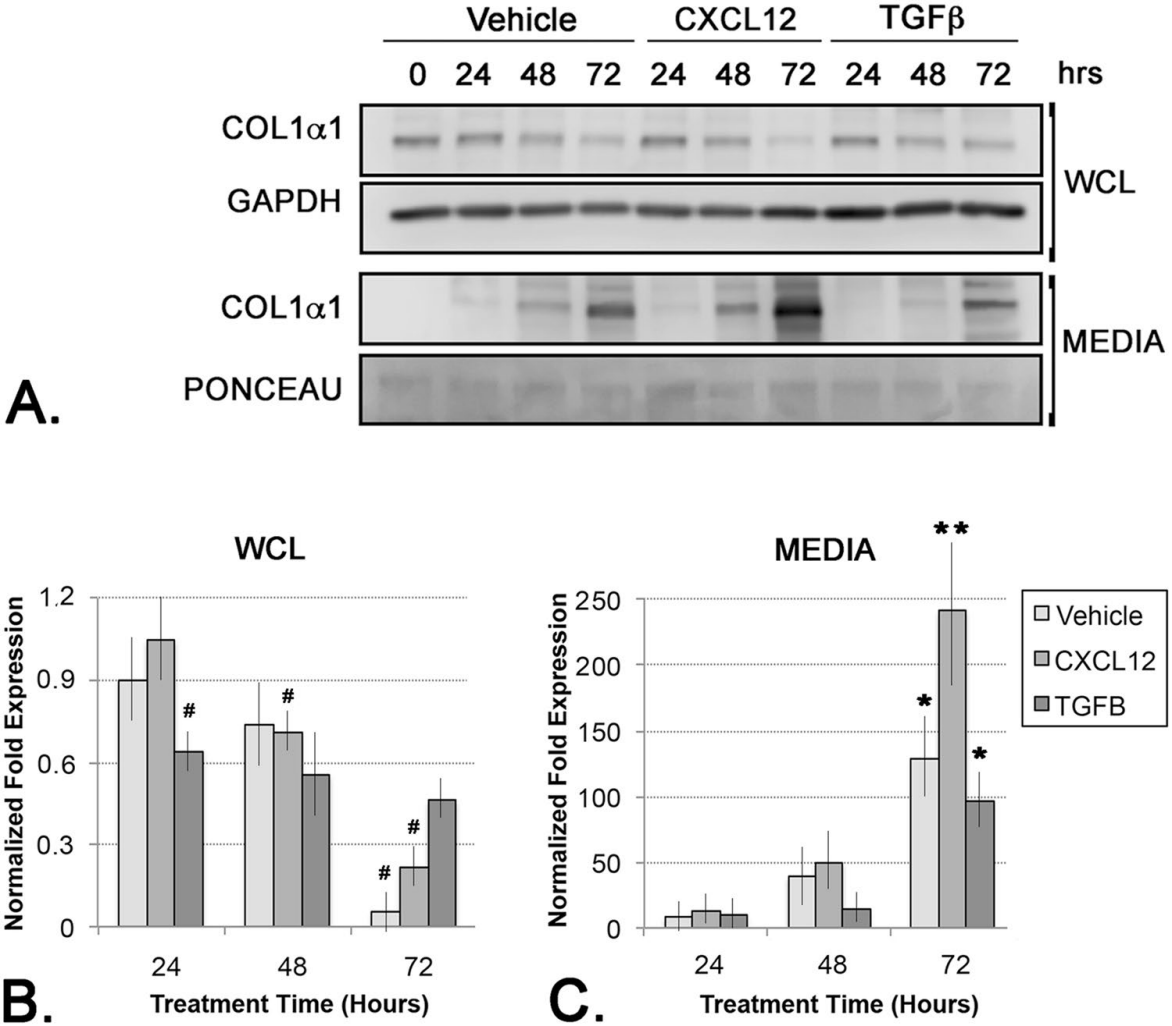
CXCL12 Up-Regulates Pro-Collagen Secretion. (**A**) Immunoblot of protein lysates (WCL) or conditioned media (MEDIA) from cells treated with vehicle, CXCL12 or TGF**β** and probed for collagen 1 (COL1**α**1) or GAPDH (as WCL loading control) proteins. Ponceau staining is shown as loading control for conditioned media immunoblot. The WCL and MEDIA blots were loaded, run, and probed separately. (**B**,**C**) Graphs of densitometric analysis of replicate immunoblots of collagen 1 protein in whole cell lysate (WCL) (**B**) or conditioned media (MEDIA) (**C**). Collagen 1 protein is significantly downregulated in WCL from CXCL12-treated cells (#) but is significantly up-regulated in MEDIA from CXCL12-treated cells compared to vehicle (**p < 0.001; ^#^ or *p < 0.05; all value shown are normalized to vehicle protein levels). Graphs represent averaged measures of triplicate experiments.

**Figure 7:**
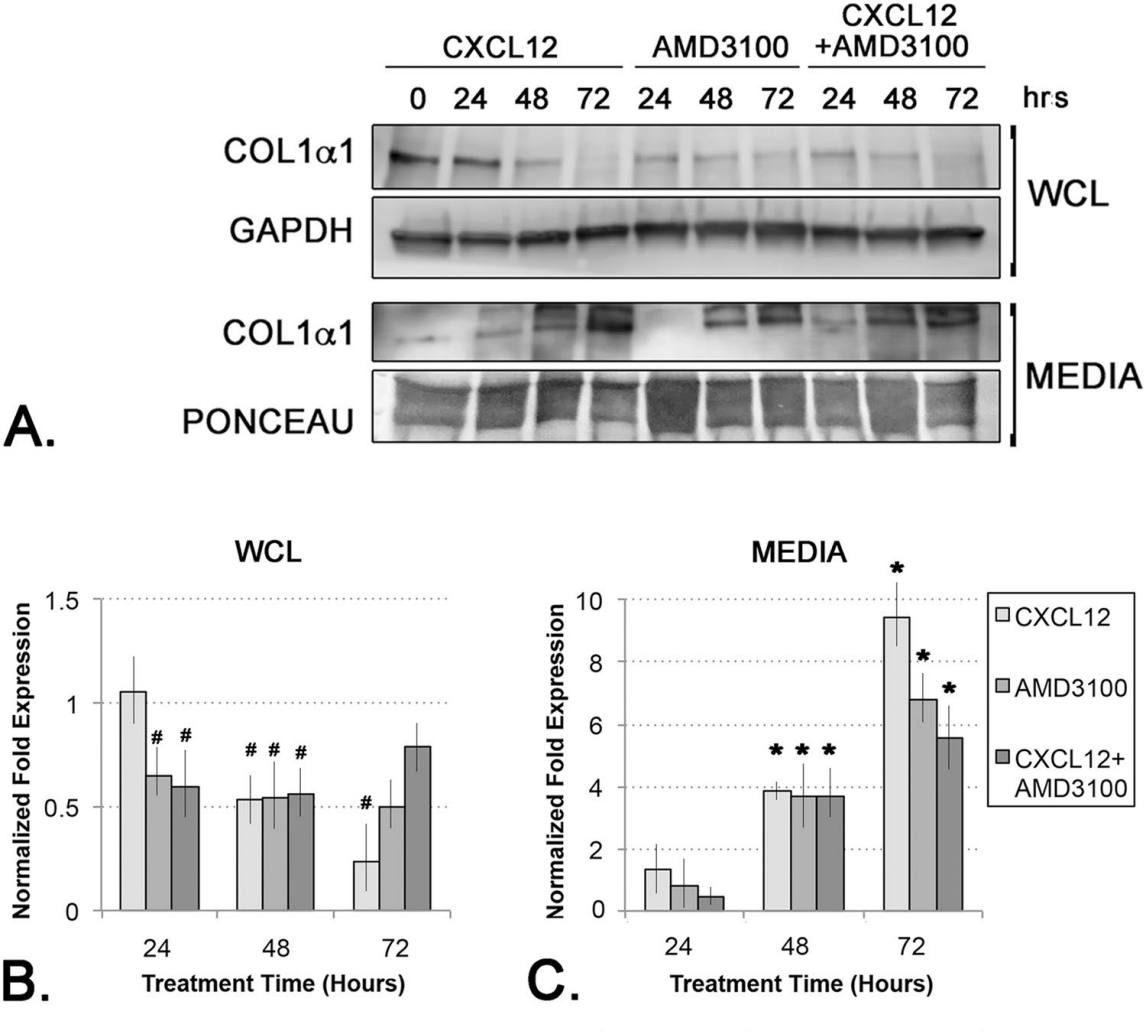
The CXCL12/CXCR4 Axis Specifically Up-Regulates Pro-Collagen Secretion. (**A**) Immunoblot of protein lysates (WCL) or conditioned media (MEDIA) from cells treated with vehicle, the small molecule CXCR4 inhibitor AMD3100, or AMD3100 + CXCL12 and probed for collagen 1 (COL1**α**1) or GAPDH (as WCL loading control) proteins. Ponceau staining is shown as loading control for conditioned media immunoblot. The WCL and MEDIA blots were loaded, run, and probed separately (**B**,**C**) Graphs of densitometric analysis of replicate immunoblots of collagen 1 protein in whole cell lysate (WCL) (**B**) or conditioned media (MEDIA) (**C**). Collagen 1 protein is significantly downregulated in WCL from AMD3100- and AMD3100 + CXCL12-treated cells (#) (**B**), and is equivalently up-regulated in all samples at 48 hrs but repressed in MEDIA from AMD3100 and AMD3100 + CXCL12-treated compared to CXCL12-treated vehicle (^#^ or *p < 0.05). Graphs represent averaged measures of triplicate experiments.

In order to assess the potential functional consequences of the observed CXCL12/CXCR4-mediated promotion of E3 ubiquitin ligase and COPII vesicle component expression, cells were next assessed for procollagen secretion levels. The cells used in these studies, N1 immortalized prostate stromal fibroblasts, constitutively express and secrete low levels of collagen, but this activity can be induced further upon stimulation with pro-fibrotic proteins such as CXCL12 or TGF**β**^3,7^. For these studies, cells were treated with CXCL12 or TGF**β**, then cellular and secreted procollagen protein levels were immunoblotted and quantitated. The results of these studies showed that cellular procollagen levels steadily decreased over a 72 hr period in all treatment groups (Fig. 6A,B). In contrast, secreted procollagen levels steadily increased over a 72 hr period in all treatment groups, and this effect was most pronounced for CXCL12-treated cells (Fig. 6A,C). CXCL12-treated cells evinced significantly higher levels of secreted procollagen than either vehicle- or TGF**β**-treated cells, which secreted similar levels of procollagen (Fig. 6A,C), suggesting that activation of the CXCL12/CXCR4 axis, but not TGF**β**/TGF**βR** axis, promoted procollagen secretion. The specificity of these cellular responses was next tested by co-treating the cells with the CXCR4 small molecule inhibitor, AMD3100. Consistent with previous data, these studies showed that cellular levels of procollagen decreased, while secreted levels concomitantly increased, consequent to CXCL12 treatment (Fig. 7A–C). Exposure to AMD3100 in the absence of CXCL12 reduced, but did not ablate, procollagen secretion, and this effect was not rescued by the addition of CXCL12 (Fig. 7A,C). These studies are consistent with a mechanism whereby procollagen secretion was coupled to CXCR4 function (Fig. 6). These data demonstrate that, in addition to promoting procollagen transcript and protein expression as previously described^3,7^, activation of the CXCL12/CXCR4 axis promotes, whereas activation of the TGF**β**/TGF**βR** axis represses, expression of key components of the CUL3-CRL complex associated with procollagen secretion and the secretion of procollagen itself.

### CXCL12/CXCR4-Mediated Up-Regulation of CUL3 is Essential for Procollagen Secretion from Prostate Fibroblasts

**Figure 8:**
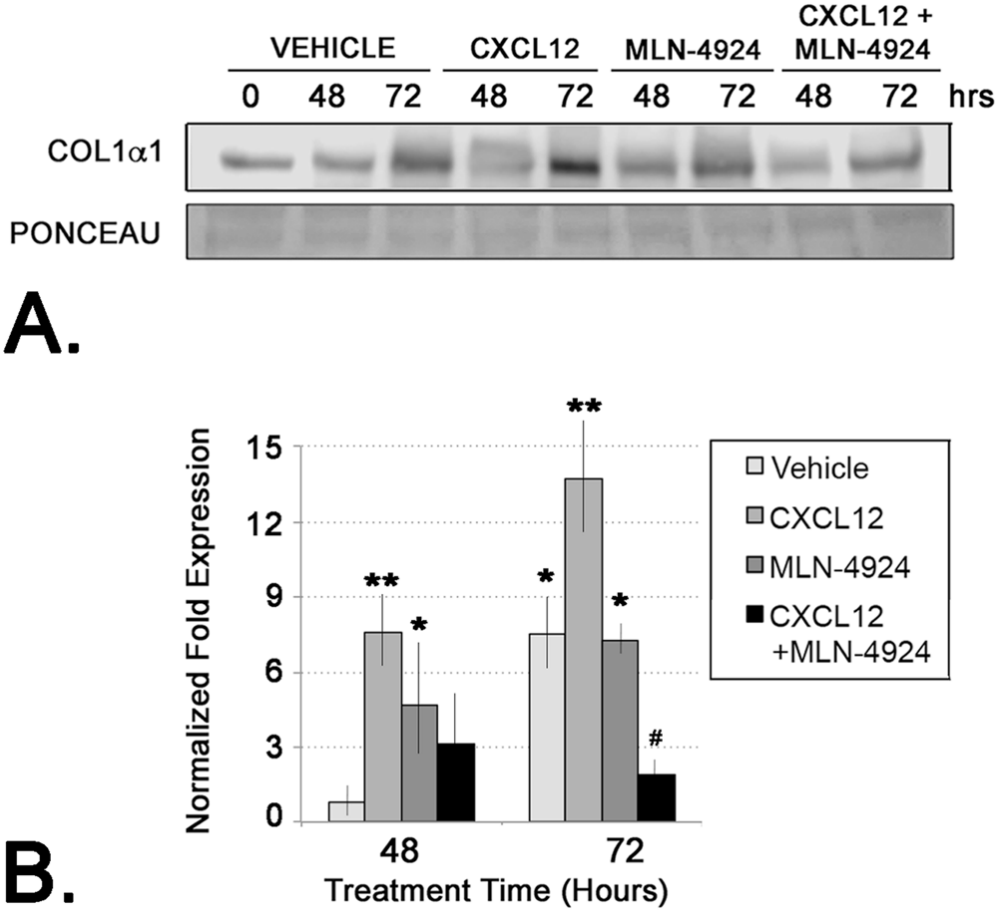
CXCL12/CXCR4-Mediated Up-Regulation of CUL3 is Essential for Procollagen Secretion from Prostate Fibroblasts. (**A**) Immunoblot of conditioned media from cells treated with vehicle, CXCL12, the small molecule cullin-RING ubiquitin E3 ligase inhibitor, MLN-4924, or CXCL12 + MLN-4924, and probed for collagen 1 (COL1**α**1). Ponceau staining is shown as loading control for conditioned media immunoblot. (**B**) Graph of densitometric analysis of triplicate immunoblots of collagen 1 protein in conditioned media after normalization to Ponceau protein. Cells treated with CXCL12 demonstrated high levels of procollagen secretion; however, treatment with MLN-4924 reduced procollagen secretion to levels at or below those observed for vehicle-treated cells, and this effect was not rescued by co-treatment with CXCL12 (^#^ or *p < 0.05). Graphs represent averaged measures of triplicate experiments.

The previously detailed studies were consistent with a mechanism involving CXCL12/CXCR axis-mediated CUL3 up-regulation associated with increased procollagen secretion. In order to test this association, cells were treated with CXCL12 in the absence or presence of MLN-4924, a cullin-RING ubiquitin E3 ligase inhibitor. This compound is an analog of adenosine 5′-monophosphate that selectively inhibits NEDD8-activating enzyme (NAE), thereby preventing neddylation and subsequent activation of cullin-RING ubiquitn E3 ligases, including CUL3^14,15^. Consistent with previous data, cells treated with CXCL12 demonstrated high levels of procollagen secretion (Fig. 8A,B). Treatment with MLN-4924 reduced procollagen secretion to levels at or below those observed for vehicle-treated cells, and this effect was not rescued by co-treatment with CXCL12 (Fig. 8A,B). These observations are consistent with a mechanism whereby CXCL12/CXCR4 axis activation promotes procollagen secretion coupled to cullin-RING ubiquitn E3 ligase activity.

## Discussion

Though unrelated in structure, both CXCL12 and TGF**β** are cytokines, are secreted proteins, and perform diverse cellular functions including, though not limited to, regulation of cellular proliferation and cellular differentiation (recently reviewed in^16,17^). Both CXCL12 and TGF**β** can promote the differentiation of multiple types of adult cells to those with a myofibroblastic phenotype^7,18^. Although both cytokines can activate MAP Kinase signaling, TGF**β** promotes myofibroblast phenoconversion in a Smad-dependent manner, whereas CXCL12/CXCR4 accomplishes this by transactivating EGFR and promoting downstream MAPK signaling^3^. These signaling events transcriptionally activate the promoters of the COL1A1 and COL1A2 genes and result in procollagen protein production, a hallmark of myofibroblast phenoconversion and extracellular matrix deposition that is characteristic of tissue fibrosis^2,4,5^.

In an effort to better understand how CXCL12 and TGF**β** cytokine activity might converge to promote fibrosis through collagen gene activation or other mechanisms, prostate stromal fibroblasts were treated with these cytokines and the resulting RNA transcriptomes analyzed. These studies demonstrated an unexpected degree of homology between CXCL12- and TGF**β**-mediated transcriptional molecular signatures and conjunction of downstream molecular functions. A distinguishing characteristic, however, was the increased association of ubiquitination/polyubiquitination and ER-to-Golgi signatures with activation of the CXCL12/CXCR4, compared to TGF**β**/TGF**β**R, axis. This signature was reflected by the observed significant depression of Cullin and KLHL12 transcript and protein levels by activation of the TGF**β**/TGF**β**R axis compared to the CXCL12/CXCR4 axis. Taken together, the data presented here suggests that activation of the TGF**β**/TGF**β**R axis inhibits, rather than promotes, COPII vesicle-mediated secretion of procollagen, which is contrary to other, overwhelmingly pro-fibrotic, activities attributed to this signaling axis. This observation may signal the existence of a previously un-described feedback or regulatory mechanism that can limit the pro-fibrotic activities of the TGF**β**/TGF**β**R axis under particular conditions.

Although the majority of the studies presented here focused on CXCL12/CXCR4-mediated up-regulation of CUL3 and its functional consequences, it should be noted that both CUL1 and CUL4A were similarly up-regulated at the RNA (not shown) and protein levels. In complex with RBX1 and SKP2, CUL1 is responsible for ubiquitylation and subsequent proteasomal degradation of p21 during the G1/S phase transition, leading to replication of DNA in advance of mitosis^19^. As noted previously, CXCL12 is a cytokine that promotes cellular proliferation of prostatic stromal fibroblast and epithelial cells^6,20^. Upregulation of Cul4A with CXCL12 was also expected due to the pro-proliferative nature of CXCL12 and the established roles of Cul4 in the cell cycle, which include chromatin remodeling and histone exchange^21^. Additional roles of Cul4 in fibrosis may be tied to Wnt/β-catenin signaling, which has been associated with fibrotic disease and of which Cul4A is a known transcriptional target^22^. Though not explored here, these CUL proteins may also have role(s) in promoting CXCL12/CXCR4-mediated cellular proliferation and myofibroblast phenoconversion in human disease.

Some caveats to the present studies should be considered. Among them is the apparent discordancy observed between CXCL12-mediated KLHL12 transcript and protein levels, i.e., although activation of the CXCL12/CXCR4 axis induced significant up-regulation of the KLHL12 transcript, a concordant up-regulation of KLHL12 protein was not observed. Although the molecular mechanism coupled to this observation was not investigated, both TGF**β** and CXCL12 are known to regulate the expression of multiple miRNAs which, in turn, depress the translation of multiple mRNAs^23,24^. Consistent with the RNASeq results reported here, it would not be surprising if activation of the TGF**β**/TGF**β**R axis compared to the CXCL12/CXCR4 axis regulated both similar and dissimilar subsets of miRNAs, and that miRNAs induced by one axis may act to depress the translation of mRNAs induced by the other axis. Further studies are required to test this hypothesis. Another concern is that, although the pro-fibrotic role(s) of CXCL12/CXCR4 activation are increasingly elucidated, it is completely unclear whether other CXC-type chemokines that also promote myofibroblast phenoconversion^7^ similarly promote procollagen secretion and ECM deposition. Second, although the coupling of CXCL12/CXCR4 activation to procollagen secretion is intriguing, many other components of the procollagen secretion pathway are not addressed by this mechanism in this or other studies. For example, CK2-mediated phosphorylation of Sec31 is required for COPII vesicle formation^25^ but there is no evidence in the current study that CK2 expression or function is regulated by CXCL12/CXCR4 interactions. Third, the role(s) of CXCR7, another cognate receptor of CXCL12 that may actually heterodimerize with CXCR4^26^, have not been delineated in many CXCL12-mediated cellular events, particularly myofibroblast phenoconversion. N1 cells express both CXCR4 and CXCR7 (Supplementary File), and the CXCR4 inhibitor, AMD3100, has been shown to act as an allosteric agonist of CXCR7 and activate EGFR^27,28^. However, we have previously shown that treatment of N1 cells with AMD3100 is associated with complete repression of EGFR activation, CXCR4- and EGFR-mediated downstream signaling, collagen expression, and myofibroblast phenoconversion^3^. Therefore, it is unlikely that potential AMD3100/CXCR7 interactions are coupled to the experimental observations reported here. Finally, although ours and other studies suggest that prostate fibroblasts behave similarly to those from other organ sites with regard to basic physiology, it cannot be discounted that tissue-specific factors may play a role in collagen secretion and myofibroblast phenoconversion.

In summary, the data presented here provide further evidence of the importance of CXCL12-mediated prostate myofibroblast phenoconversion as a contributing factor to collagen deposition and augmented ECM formation characteristic of tissue fibrosis. Moreover, these studies identify a previously un-described mechanism whereby activation of the CXCL12/CXCR4 axis increases CUL3 protein levels and procollagen secretion, a mechanism that is opposed by activation of the TGF**β**-TGF**β**R axis. These findings suggest that multiple mechanisms may be active within tissues undergoing aberrant wound-healing responses; that these mechanisms may be differently regulated by the medley of pro-fibrotic proteins within the tissue microenvironment, and that different tissues may be subject to all or a subset of these mechanisms. Elucidation of the mechanisms specifically promoted, or repressed, by particular pro-fibrotic proteins may be required in order to identify suitable therapeutic targets for the management or prevention of fibrosis in different tissue types and organ sites.

## Materials and Methods

### Cell Culture and Treatment

All experiments were performed in N1 cells which were derived from a stromal nodule of benign prostatic hyperplasia, exhibit a fibroblastic morphology, express fibroblastic markers vimentin and calponin, and demonstrate secretion and proliferation profiles consistent with aging primary prostate fibroblasts^6^,^7^. N1 fibroblasts were plated at 10,000 cells/cm^2^ in 5% HIE media (Ham’s F-12, 5% FBS, Insulin [5 μg/mL], EGF [10 ng/mL], Hydrocortisone [1 μg/mL], Fungizone [0.5 μg/mL], Gentamicin [0.05 mg/mL]), and starved for 24 hours in serum free (SF HIE) media (Ham’s F12, EGF [50 ng/mL], 0.1% BSA, Insulin [5 μg/mL], Transferrin [5 μg/mL], 50 μM sodium selenite, 10 uM 3,3′,5-triiodo-L-thyronine, Hydrocortisone [1 μg/mL], Fungizone [0.5 μg/mL], Gentamicin [0.05 mg/mL]) prior to treatment. Starved fibroblasts were washed with PBS, then treated with 100pM recombinant human CXCL12 (R&D Systems) or 0.01% BSA vehicle, and/or with 4 ng/mL activated recombinant human TGFβ (TGF**β**–1, R&D Systems) or 20 nM citrate vehicle in fresh SF media, then incubated until collection at the desired time points. For inhibitor treatments, cells were pre-incubated with the CXCR4-specific small molecular inhibitor, AMD3100 (25 uM, Sigma-Aldrich), the TGF**β**R1 small molecular inhibitor, SB431542 (500 nM, Sigma-Aldrich), or the cullin-RING ubiquitin E3 ligase (CUL3) inhibitor MLN-4924 (100 nM, Active Biochem), in fresh media for 2 hours prior to CXCL12 or TGFΒ treatment.

### RNA Extraction and Gene Expression Analysis

N1 cells were grown to 70% confluence in 6-cm tissue culture plates. RNA was isolated by phenol-chloroform extraction using Trizol reagent (Invitrogen, Carlsbad, CA). RNA was assessed for purity by A260/A280 ratio and quantified using a Nanodrop spectrophotometer. Following quantification, 1 ug of RNA was reverse transcribed using a High Capacity cDNA Reverse Transcription Kit (Applied Biosystems, Carlsbad, CA). qRT-PCR was performed using a QuantStudio 12 K Flex Real-Time PCR System, reagents and software (Applied Biosystems, Carlsbad, CA). Reactions were performed in triplicate, including no template controls and amplification of an endogenous control transcript, Larger Ribosomal Protein (RPLPO), to assess template concentration and loading precision. Cycle number to threshold was calculated for each gene by subtracting the average control value from each average experimental value and normalized to RPLPO (loading control) using the Pfaffl method. Molecular probes used were Hs01117001_m1 (CUL3), Hs00603108_mH (KLHL12), Hs0016400_m1 (COL1α1), and Hs99999902_m1 for RPLPO (Applied Biosystems, Carlsbad, CA).

### Library Preparation and Sequencing

Libraries were prepared from RNA isolated from N1 cells treated with vehicle, CXCL12 or TGF**β** as described above. Total RNA samples were DNAse-treated and 300 ng was used as input to create single indexed (6 base pairs) RNA-Seq libraries using the TruSeq RNA preparation kit according to the manufacturer’s protocol (Illumina, Carlsbad, CA). Samples were pooled together and sequenced on a two-lane flow cell using on-board cluster generation with the Illumina HiSeq^TM^ 2500 instrument. A 51 cycle paired-end rapid run of the single indexed RNA-Seq libraries was performed. Raw data of the Bcl base call files were then de-multiplexed upon completion.

### Bioinformatics Analysis

Bcl basecall files were converted to FASTQ format using Illumina’s bcl2fastq conversion tool v1.8.4. Stringent conversion parameters were set by not allowing a mismatch in the index during the demultiplexing process. Additionally, FASTQ files were created by overwriting the default parameter to split the files after 4 million reads, generating a single FASTQ file for each sample replicate. Alignment of reads were carried out using Tophat v2.0.14 post FASTQ file conversion using Illumina’s bcl2fastq tool v1.8.4^29^. Alignment parameters for Tophat consisted of utilizing the default parameters, which were sufficient for downstream analysis of data. Cufflinks v2.2.1 was used to estimate the relative abundance of transcripts aligned for all the samples. Cuffdiff v2.2.1 was used to perform the differential gene expression analysis post quantification of the transcripts for the samples^30^. Differential gene expression (DGE) analysis dgeR analysis was performed using the Empirical Analysis of Digital Gene Expression Data in R (edgeR), and a statistical cutoff was set to p-value ≤ 0.001. The edgeR table is provided as Supplementary Dataset. Venn diagrams were generated using the *overlapper* and *vennPlot* functions in R/Bioconductor.

Gene set enrichment analysis using Pathview v1.10.1 was used to elucidate the top cellular pathways that are overall up-regulated, or down-regulated in vehicle, CXCL12, or TGF**β** treated cells^32^. In addition, selected pathways of interest were also picked for visualization of the overall regulation of the pathways. The statistical cutoff was set to a q-value ≤0.05. Network Analysis of the differential expression analysis was accomplished using Cytoscape v3.3.0^33^.

Gene ontology analysis of the gene sets were performed using ClueGO v2.2.3 and GOstats v2.36.0^34,35^. Log odds ratios were calculated and statistical cutoffs for all analyses were set to a p-values of ≤0.05.

### Immunoblotting

Cells were collected in protease inhibitor cocktail (PIC)-containing PBS and lysed in RadioImmunoPrecipitation Assay (RIPA) Buffer (10 mM Tris-Cl (pH 8.0), 1 mM EDTA, 0.5 mM EGTA, 1% Triton X-100, 0.1% sodium deoxycholate, 0.1% SDS, 140 mM NaCl). Protein was quantified via Bradford assay with Bio-Rad OneStep Bradford reagent and an Elx800 Microplate Reader (Bio-Tek) with Gen5 software, and all samples standardized to 15ug of total protein. Prior to electrophoresis, lysates were combined with 4× Lithium Dodecyl Sulfate (LDS) Sample Buffer and β-ME. Samples were run in 8% Tris-Glycine precast gels (Life Technologies), then transferred to nitrocellulose membranes (Thermo Scientific) using the Pierce G2 blotter. Membranes were blocked using a 5% Milk TBS-T solution for one hour. Primary antibody incubation was performed overnight at 4 °C in 5% BSA TBS-T (50 mM Tris, 150 mM NaCl, 0.05% Tween 20, pH 7.6) solution containing monoclonal rabbit antibodies against COL1α1 (ab34710) from AbCam, CUL1 (#4995), CUL3 (#2759), CUL4A (#2699), KLHL12 (#9406) and GAPDH (#2118), from Cell Signaling Technologies.

Conditioned media samples were concentrated by centrifugal filtration at 3,000 × g for 40 minutes using 100 K MWCO Amicon Ultra filter units. 5 uL volumes of each conditioned media sample were electrophoresed with 4× LDS Buffer without β-ME. After transfer, nitrocellulose membranes were stained with 0.1% Ponceau-S (0.1% Ponceau S in 5.0% acetic acid, Sigma #P7170) and photographed for use as a loading control, then blotted as above with primary antibody against COL1α1 (ab34710) from AbCam. Secondary incubations using Horse Radish Peroxidase (HRP) conjugated anti-rabbit antibody [1:5,000 dilution, Cell Signaling] in TBS-T were performed for 1 hour at room temperature for all samples. Membranes were washed twice with TBS-T between each incubation. After blotting, membranes were incubated in chemiluminescent substrate and scanned using the Odyssey c-Digit (LI-COR), for the detection of HRP-conjugated antibodies. Immunoblots were quantified by pixel density quantification and analyzed using the ImageStudio software suite (LI-COR).

All immunoblot studies were conducted in replicate (duplicate or triplicate); representative individual experiments from these replicate sets are shown in the manuscript. All lanes in all immunoblots were loaded contiguously; individual immunblot filters were stripped and re-probed sequentially as described in the Figure Legends.

### Statistical Analysis

Averages and standard deviations were calculated and compared using student’s t-tests. In all tests, p < 0.05 was considered statistically significant. Other bioinformatics-based statistical analyses were conducted as detailed above.

### Data Availability Statement

The edgeR dataset file is supplied as Supplementary Dataset. The N1 cell line is available upon request. All other reagents are commercially available.

### Third Party Rights

Figure 4B is provided Modified from^13^ and used with permission from Elsevier Limited (VAT number GB 494 6272 12). This has been appended to the cover letter.

## Electronic supplementary material

Supplementary Information

Supplementary Dataset 1
