# Supplementary material for: CXCL12/CXCR4-Mediated Procollagen Secretion Is Coupled To Cullin-RING Ubiquitin Ligase Activation
Source: Sci Rep. 2018 Feb 22;8:3499. doi: 10.1038/s41598-018-21506-7 (PMC5823879; doi:10.1038/s41598-018-21506-7)
Supplement: Supplementary file 1 — Supplementary Information [file 41598_2018_21506_MOESM1_ESM.pdf]

CXCL12/CXCR4-Mediated Procollagen Secretion Is Coupled To Cullin-RING Ubiquitin Ligase Activation

Susan Patalano[1,2], José Rodríguez-Nieves[2], Cory Colaneri[1], Justin Cotellessa[1,2], Diego Almanza[1,2], Alisa Zhilin-Roth[1,2], Todd Riley[1], Jill Macoska[1,2]

[1]Department of Biology, University of Massachusetts Boston, [2]Center for Personalized Cancer Therapy, University of Massachusetts Boston

Supplementary File 2

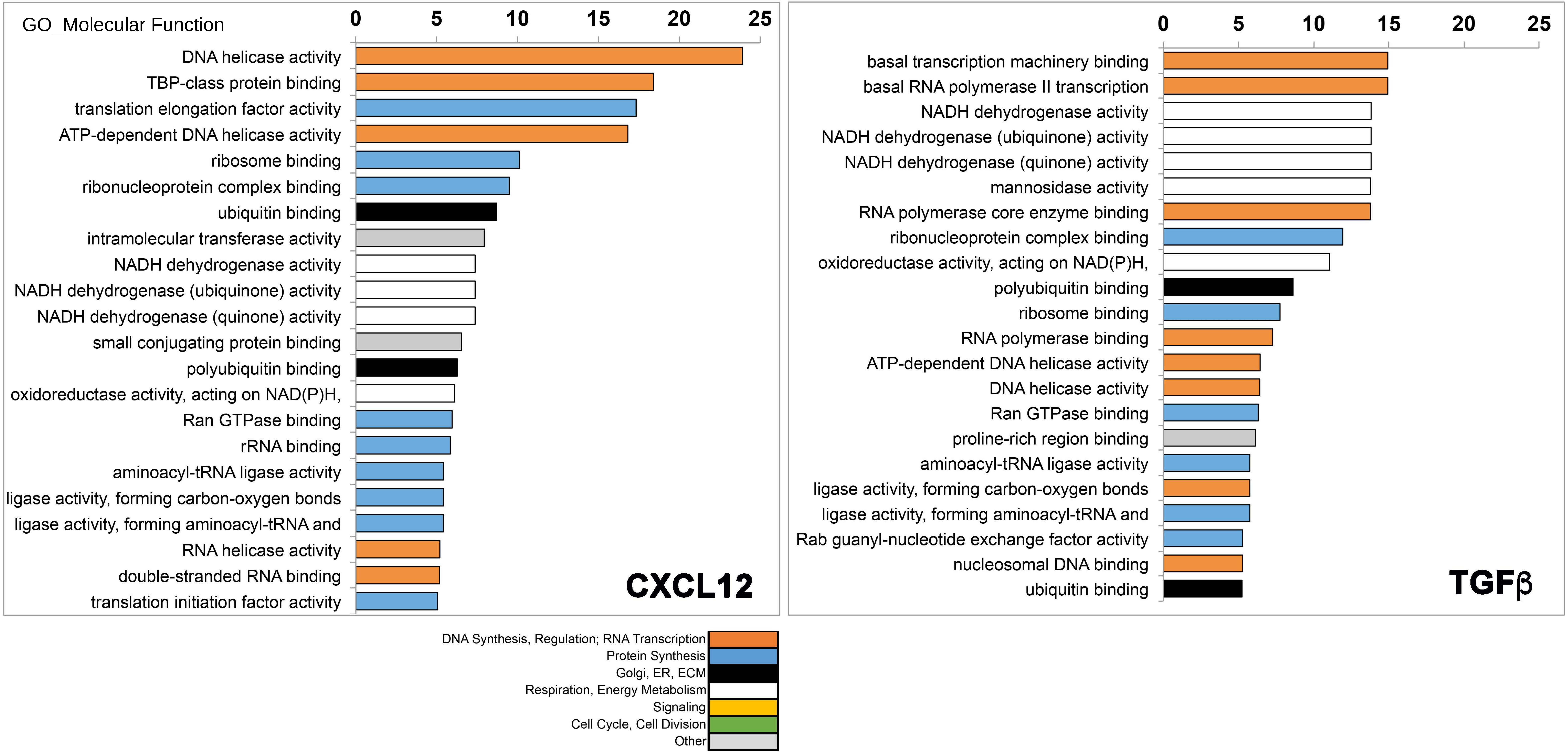
GO_Molecular Function
0 5 10 15 20 25
DNA helicase activity
TBP-class protein binding
translation elongation factor activity
ATP-dependent DNA helicase activity
ribosome binding
ribonucleoprotein complex binding
ubiquitin binding
intramolecular transferase activity
NADH dehydrogenase activity
NADH dehydrogenase (ubiquinone) activity
NADH dehydrogenase (quinone) activity
small conjugating protein binding
polyubiquitin binding
oxidoreductase activity, acting on NAD(P)H,
Ran GTPase binding
rRNA binding
aminoacyl-tRNA ligase activity
ligase activity, forming carbon-oxygen bonds
ligase activity, forming aminoacyl-tRNA and
RNA helicase activity
double-stranded RNA binding
translation initiation factor activity
CXCL12
0 5 10 15 20 25
basal transcription machinery binding
basal RNA polymerase II transcription
NADH dehydrogenase activity
NADH dehydrogenase (ubiquinone) activity
NADH dehydrogenase (quinone) activity
mannosidase activity
RNA polymerase core enzyme binding
ribonucleoprotein complex binding
oxidoreductase activity, acting on NAD(P)H,
polyubiquitin binding
ribosome binding
RNA polymerase binding
ATP-dependent DNA helicase activity
DNA helicase activity
Ran GTPase binding
proline-rich region binding
aminoacyl-tRNA ligase activity
ligase activity, forming carbon-oxygen bonds
ligase activity, forming aminoacyl-tRNA and
Rab guanyl-nucleotide exchange factor activity
nucleosomal DNA binding
ubiquitin binding
TGFβ
DNA Synthesis, Regulation; RNA Transcription
Protein Synthesis
Golgi, ER, ECM
Respiration, Energy Metabolism
Signaling
Cell Cycle, Cell Division
Other

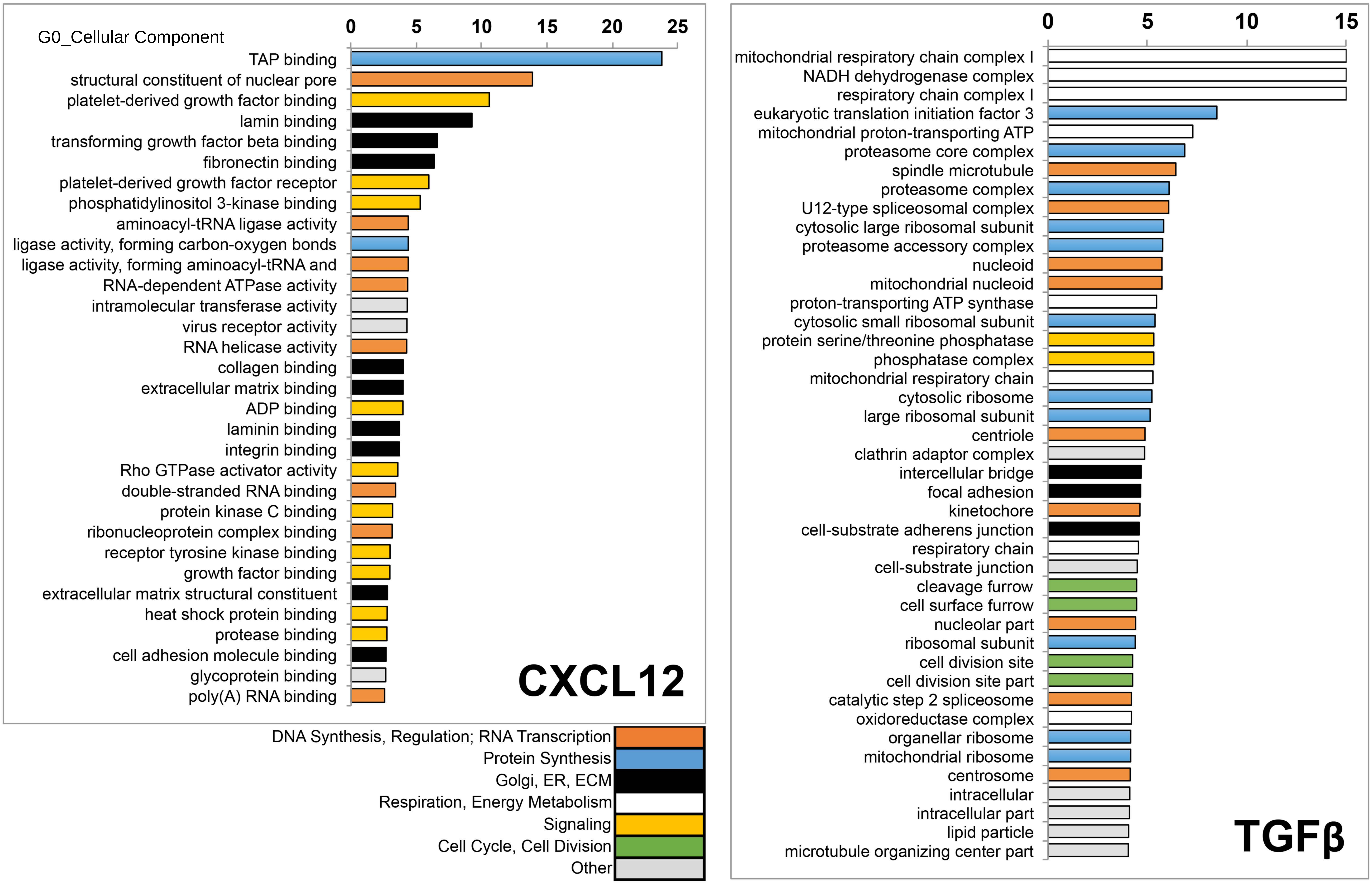
G0_Cellular Component
0
5
10
15
20
25
TAP binding
structural constituent of nuclear pore
platelet-derived growth factor binding
lamin binding
transforming growth factor beta binding
fibronectin binding
platelet-derived growth factor receptor
phosphatidylinositol 3-kinase binding
aminoacyl-tRNA ligase activity
ligase activity, forming carbon-oxygen bonds
ligase activity, forming aminoacyl-tRNA and
RNA-dependent ATPase activity
intramolecular transferase activity
virus receptor activity
RNA helicase activity
collagen binding
extracellular matrix binding
ADP binding
laminin binding
integrin binding
Rho GTPase activator activity
double-stranded RNA binding
protein kinase C binding
ribonucleoprotein complex binding
receptor tyrosine kinase binding
growth factor binding
extracellular matrix structural constituent
heat shock protein binding
protease binding
cell adhesion molecule binding
glycoprotein binding
poly(A) RNA binding
CXCL12
DNA Synthesis, Regulation; RNA Transcription
Protein Synthesis
Golgi, ER, ECM
Respiration, Energy Metabolism
Signaling
Cell Cycle, Cell Division
Other
0
5
10
15
mitochondrial respiratory chain complex I
NADH dehydrogenase complex
respiratory chain complex I
eukaryotic translation initiation factor 3
mitochondrial proton-transporting ATP
proteasome core complex
spindle microtubule
proteasome complex
U12-type spliceosomal complex
cytosolic large ribosomal subunit
proteasome accessory complex
nucleoid
mitochondrial nucleoid
proton-transporting ATP synthase
cytosolic small ribosomal subunit
protein serine/threonine phosphatase
phosphatase complex
mitochondrial respiratory chain
cytosolic ribosome
large ribosomal subunit
centriole
clathrin adaptor complex
intercellular bridge
focal adhesion
kinetochore
cell-substrate adherens junction
respiratory chain
cell-substrate junction
cleavage furrow
cell surface furrow
nucleolar part
ribosomal subunit
cell division site
cell division site part
catalytic step 2 spliceosome
oxidoreductase complex
organellar ribosome
mitochondrial ribosome
centrosome
intracellular
intracellular part
lipid particle
microtubule organizing center part
TGFβ

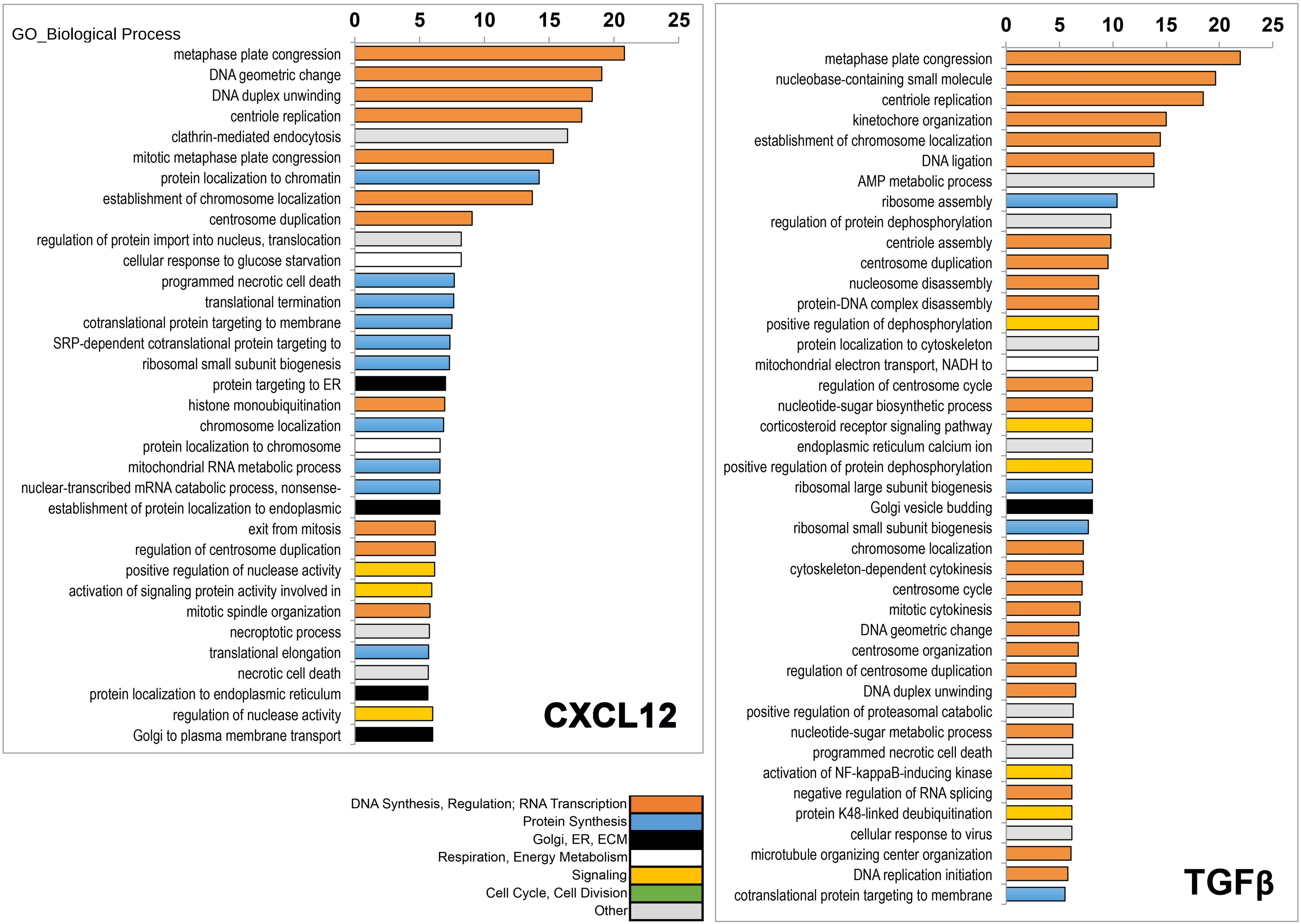

GO_Biological Process
0
5
10
15
20
25
metaphase plate congression
DNA geometric change
DNA duplex unwinding
centriole replication
clathrin-mediated endocytosis
mitotic metaphase plate congression
protein localization to chromatin
establishment of chromosome localization
centrosome duplication
regulation of protein import into nucleus, translocation
cellular response to glucose starvation
programmed necrotic cell death
translational termination
cotranslational protein targeting to membrane
SRP-dependent cotranslational protein targeting to
ribosomal small subunit biogenesis
protein targeting to ER
histone monoubiquitination
chromosome localization
protein localization to chromosome
mitochondrial RNA metabolic process
nuclear-transcribed mRNA catabolic process, nonsense-
establishment of protein localization to endoplasmic
exit from mitosis
regulation of centrosome duplication
positive regulation of nuclease activity
activation of signaling protein activity involved in
mitotic spindle organization
necroptotic process
translational elongation
necrotic cell death
protein localization to endoplasmic reticulum
regulation of nuclease activity
Golgi to plasma membrane transport
CXCL12
DNA Synthesis, Regulation; RNA Transcription
Protein Synthesis
Golgi, ER, ECM
Respiration, Energy Metabolism
Signaling
Cell Cycle, Cell Division
Other
metaphase plate congression
nucleobase-containing small molecule
centriole replication
kinetochore organization
establishment of chromosome localization
DNA ligation
AMP metabolic process
ribosome assembly
regulation of protein dephosphorylation
centriole assembly
centrosome duplication
nucleosome disassembly
protein-DNA complex disassembly
positive regulation of dephosphorylation
protein localization to cytoskeleton
mitochondrial electron transport, NADH to
regulation of centrosome cycle
nucleotide-sugar biosynthetic process
corticosteroid receptor signaling pathway
endoplasmic reticulum calcium ion
positive regulation of protein dephosphorylation
ribosomal large subunit biogenesis
Golgi vesicle budding
ribosomal small subunit biogenesis
chromosome localization
cytoskeleton-dependent cytokinesis
centrosome cycle
mitotic cytokinesis
DNA geometric change
centrosome organization
regulation of centrosome duplication
DNA duplex unwinding
positive regulation of proteasomal catabolic
nucleotide-sugar metabolic process
programmed necrotic cell death
activation of NF-kappaB-inducing kinase
negative regulation of RNA splicing
protein K48-linked deubiquitination
cellular response to virus
microtubule organizing center organization
DNA replication initiation
cotranslational protein targeting to membrane
TGFβ

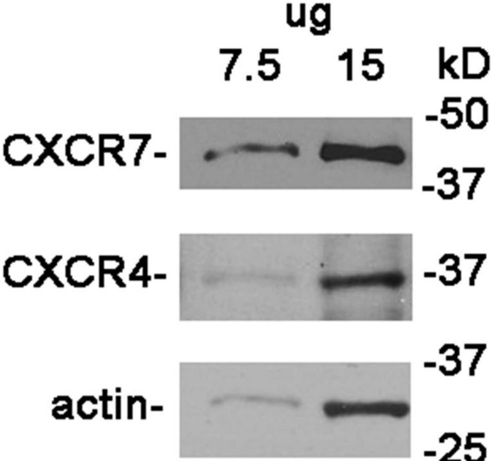
ug
7.5
15
kD
-50
-37
-37
-37
-25
CXCR7-
CXCR4-
actin-
